# Au–Ag assembled on silica nanoprobes for visual semiquantitative detection of prostate-specific antigen

**DOI:** 10.1186/s12951-021-00817-4

**Published:** 2021-03-12

**Authors:** Hyung-Mo Kim, Jaehi Kim, Jaehyun An, Sungje Bock, Xuan-Hung Pham, Kim-Hung Huynh, Yoonsik Choi, Eunil Hahm, Hobeom Song, Jung-Won Kim, Won-Yeop Rho, Dae Hong Jeong, Ho-Young Lee, Sangchul Lee, Bong-Hyun Jun

**Affiliations:** 1 Department of Bioscience and Biotechnology, Konkuk University, Seoul, Korea; 2 Department of Chemistry Education, Seoul National University, Seoul, Korea; 3 BioSquare Inc, Seongnam, Korea; 4 School of International Engineering and Science, Jeonbuk National University, Jeonju, Korea; 5 Department of Nuclear Medicine, Seoul National University Bundang Hospital, Seongnam, Korea; 6 Department of Urology, Seoul National University Bundang Hospital, Seongnam, Korea

**Keywords:** Prostate-specific antigen, Lateral-flow immunoassay, Nanoparticle, Prostate cancer, Colorimetric assay, Colloid gold nanoparticle

## Abstract

**Background:**

Blood prostate-specific antigen (PSA) levels are widely used as diagnostic biomarkers for prostate cancer. Lateral-flow immunoassay (LFIA)-based PSA detection can overcome the limitations associated with other methods. LFIAbased PSA detection in clinical samples enables prognosis and early diagnosis owing to the use of high-performance signal reporters.

**Results:**

Here, a semiquantitative LFIA platform for PSA detection in blood was developed using Au–Ag nanoparticles (NPs) assembled on silica NPs (SiO2@Au–Ag NPs) that served as signal reporters. Synthesized SiO2@Au–Ag NPs exhibited a high absorbance at a wide wavelength range (400–800 nm), with a high scattering on nitrocellulose membrane test strips. In LFIA, the color intensity of the test line on the test strip differed depending on the PSA concentration (0.30–10.00 ng/mL), and bands for the test line on the test strip could be used as a standard. When clinical samples were assessed using this LFIA, a visual test line with particular color intensity observed on the test strip enabled the early diagnosis and prognosis of patients with prostate cancer based on PSA detection. In addition, the relative standard deviation of reproducibility was 1.41%, indicating high reproducibility, and the signal reporter showed good stability for 10 days.

**Conclusion:**

These characteristics of the signal reporter demonstrated the reliability of the LFIA platform for PSA detection, suggesting potential applications in clinical sample analysis.

**Supplementary Information:**

The online version contains supplementary material available at 10.1186/s12951-021-00817-4.

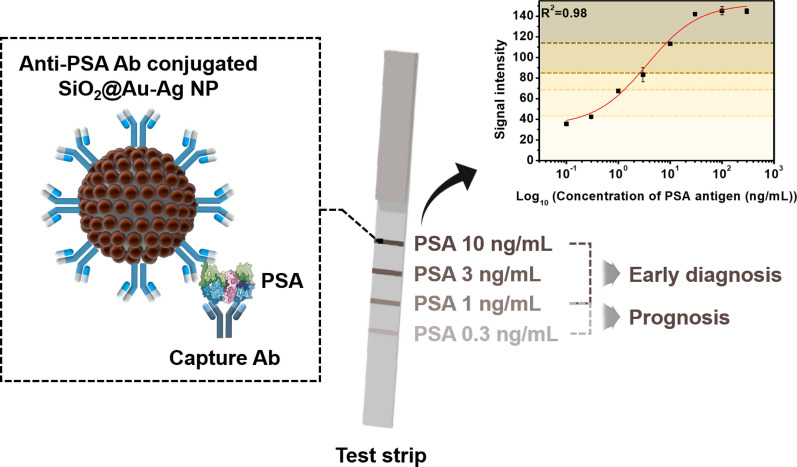

## Background

Prostate cancer (PCa) is the second most common cancer in men worldwide and the fifth leading cause of cancer-related death [1]. PCa can be cured by radical prostatectomy or radiation treatment (RT) during early stages; thus, early diagnosis is very important [2]. As the levels of prostate-specific antigen (PSA) increase after the onset of PCa, PSA is one of the most commonly used biomarkers for the diagnosis of PCa at the early stages, thereby reducing the mortality rates [3]. Based on the PSA levels, the following diagnoses are made: PSA ≤ 2.50 ng/mL, normal; PSA of 4.00–10.00 ng/mL, early stage PCa; PSA ≥ 10 ng/mL, advanced stage [4]. In patients with 2.50–4.00 ng/mL PSA, the status of PCa is not clear, and studies have emphasized that re-adjustment of the cutoff value for PSA levels may be necessary [5–8]. Additionally, after radical prostatectomy—the standard treatment for localized PCa—measurement of serum PSA levels is important for monitoring PCa recurrence [9]. In particular, in previous studies, a cutoff value of 0.30 ng/mL PSA after curative treatment was suggested for predicting biochemical recurrence (BCR) after RT [10]. Accordingly, PSA levels after radical prostatectomy should be continuously examined because the measurement of PSA levels after surgery is a relatively simple approach for diagnosing BCR [11, 12]. However, healthy men who undergo PCa screening and patients with PCa who receive treatment and need close follow-up based on their PSA levels are forced to undergo many expensive tests, resulting in a high burden [13–15]. Therefore, the development of an inexpensive, simple method for PSA measurement that is suitable for various applications, including assessment of prognosis, is essential.

Enzyme-linked immunosorbent assay (ELISA)—one of the most commonly used methods to detect PSA—is often used for the identification of biomarkers owing to its specificity, reproducibility, quantitative nature, and low detection limit (pg/mL to fg/mL level) [16]. However, PSA analysis using ELISA is time- and cost-intensive and requires well-trained technicians [17]. Lateral-flow immunoassay (LFIA)—which is based on test strips—has been widely recognized as an alternative to ELISA, owing to its economical and rapid analysis of biomolecules, such as PSA. LFIA is a low-cost, simple approach that can be employed on a large scale for clinical applications [18]. Moreover, LFIA enables visualized qualitative or semiquantitative analysis within 30 min [17]. For sensitive detection, optical nanoparticles, such as colloid gold nanoparticles (AuNPs) or silver nanoparticles (AgNPs), which are easy to prepare and exhibit strong optical reactions and affinity for antibodies during conjugation are widely used in LFIA [19–21]. In particular, AuNPs are most often employed with LFIA for qualitative and semiquantitative analyses [22]. These AuNPs have several advantages, including a high extinction cross-section, colloidal stability, and affinity for antibodies in the visible spectrum as evidenced using surface plasmon resonance [23]. In LFIA, signal reporters, such as AuNPs conjugated to the detection antibody, are captured on the test line of the LFIA nitrocellulose (NC) membrane together with the target, thereby enabling visualization of the result. When colloid AuNPs were used as signal reporters for detecting PSA in LFIA, PSA detection was confirmed visually up to 0.50–3.00 ng/mL [24–27]. Although biosensing is the primary use of AuNPs, many methods based on core/shell-type NPs, including conventional AuNPs for enabling the detection of PSA signals with high sensitivity in LFIA have been reported. Xia et al. designed a colorimetric LFIA platform using core/shell-type Au@Pt NPs as signal reporters in low-sensitivity mode and color-catalyzed Au@Pt NPs as signal reporters using a color molecule in the high-sensitivity mode for the detection of PSA [25]. Under optimized conditions, the naked eye detection limits of low- and high-sensitivity modes were 2 ng/mL and 20 pg/mL, respectively. However, detection of various concentrations of PSA using this LFIA platform is a complicated procedure owing to the separation of the low- and high-sensitivity modes. López et al. developed silver and gold enhancement methods for LFIA, which enabled simple detection of PSA by the naked eye [24]. Under optimized conditions, the naked-eye detection limit was 0.1 ng/mL when silver-enhanced AuNPs were used; this was five times lower than that for existing AuNPs. However, this method exhibits nonspecific binding in LFIA, and as it is difficult to handle samples via centrifugation, obtaining 100% recovery following antibody introduction is challenging.

In LFIA, the conditions used for the signal reporter should be efficient and reproducible, and there should be no nonspecific binding [28]. Signal reporters based on metal NPs assembled on silica NPs could overcome the problems of efficiency, reproducibility, and binding specificity owing to the ease of handling and surface modulation. The sizes of silica NPs can also be easily modulated, and modification of the surface is possible, thereby enabling the metal NPs to be introduced on the surface of silica NP [29]. Moreover, metal NP assembly on silica NPs could result in strong localized surface plasmon resonance (LSPR) compared with single NPs [29]. Among metal NPs, Au–Ag alloy NPs exhibit stronger LSPR than single AuNPs or AgNPs and show various colors when the AuNP to AgNP ratio is altered [30, 31]. Based on these advantages of Au–Ag alloy NPs, our group recently reported nanostructures with Au–Ag NPs clustered on a silica core [31–33]. These nanostructures had a single broad absorbance peak, and the formation of optimized Au–Ag alloy NPs showed distinct colors depending on the target concentration [34, 35].Accordingly, in this study, we developed an LFIA platform using Au–Ag alloy NPs assembled on silica NPs (SiO_2_@Au–Ag NPs) for the visual analysis of PSA. SiO_2_@Au–Ag NPs were synthesized using an optimized synthesis method. Various concentrations of PSA were detected based on the color intensity of the test line in the test strip. By using this result as a standard, it was possible to semi-quantitatively detect PSA concentrations corresponding to those associated with early diagnosis and prognosis in the clinical samples. In addition, this LFIA platform with SiO_2_@Au–Ag NPs was stable exhibited high reproducibility in terms of results for 10 days with signal intensity.

## Materials and methods

### Materials

Tetraethyl orthosilicate (TEOS), 3-aminopropyltriethoxysilane (APTS), silver nitrate (AgNO_3_), chloroauric acid (HAuCl_4_), ascorbic acid (AA), polyvinylpyrrolidone (wt. 10,000, PVP), sodium borohydride (NaBH_4_), succinic anhydride, 11-mercaptoundecanoic acid (MUA), *N*-(3-dimethylaminopropyl)-*N′*-ethylcarbodiimide (EDC) hydrochloride, *N*-hydroxysulfosuccinimide (Sulfo-NHS) sodium salt, 2-(*N*-Morpholino)ethanesulfonic acid hydrate (MES hydrate), phosphate-buffered saline (PBS, pH 7.4), Tween 20, and ethanolamine were purchased from Sigma-Aldrich (St. Louis, MO, USA). Aqueous ammonium hydroxide (NH_4_OH, 27%) and 1-methyl-2-pyrrolidinone (NMP) were purchased from Daejung (Siheung, Korea). The backing card, nitrocellulose (NC) membrane, absorbent pad, cassette, monoclonal anti-PSA antibody (mouse, anti-PSA Ab; cat. nos. 14801 and 14,803), and goat anti-rabbit IgG Ab were purchased from Bore Da Biotech Co. Ltd. (Seongnam, Korea).

### Synthesis of SiO_2_@Au–Ag NPs

The SiO_2_@Au–Ag NPs were synthesized as previously reported [36]. First, the SiO_2_ NPs (approximately 160 nm in diameter) were synthesized using the Stöber method [37]. The mixture containing of TEOS (1.6 mL) and NH_4_OH (3 mL) in ethanol (40 mL) was stirred for 20 h at 25 °C. The mixture was centrifuged at 8500 rpm for 15 min, and particles were washed several times using EtOH. For introducing the amine group onto the surface of silica NPs, the mixture containing SiO_2_ NPs (200 mg), absolute EtOH (4 mL), APTS (200 µL), and NH_4_OH (40 µL) was mixed and stirred for 12 h at 25 °C. Next, the mixture was centrifuged at 8500 rpm for 15 min and washed several times using EtOH. For preferentially introducing AuNPs (7 nm) onto the surface of aminated SiO_2_ NPs (SiO_2_@Au NPs), AuNPs were synthesized using HAuCl_4_ and NaBH_4_ for 12 h with gentle shaking at 25 °C. Consequently, AuNPs (7 nm in diameter), which had negatively charged ligands on their surfaces, were obtained after washing with centrifugation. Subsequently, aminated SiO_2_ NPs (1 mg) resuspended in EtOH were mixed with AuNPs (1 mM) in deionized water, and the mixture was sonicated for 30 min and incubated overnight. The mixture was centrifuged at 8500 rpm for 15 min and washed several times using EtOH. Thereafter, to create an Ag shell on the surface of SiO_2_ NPs, the NPs (200 μg) were dispersed in 9.8 mL water containing 20 μL of 10 mM ascorbic acid and 10 mg polyvinylpyrrolidone. This mixture was then stirred after the addition of 10 mM AgNO_3_ (20 µL) for 15 min at 25 °C to reduce Ag^+^ ions to Ag. Next, the reduction steps were repeated with 300 µM AgNO_3_ (20 µL). The mixture was centrifuged at 8500 rpm for 15 min, washed several times using EtOH, and dispersed in EtOH.

### Conjugation of anti-PSA Ab onto the surface of SiO_2_@Au–Ag NPs

For introducing the carboxyl groups onto the surface, SiO_2_@Au–Ag NPs reconstituted in EtOH (200 μg/mL, 1 mL) were mixed with 2 mM mercaptoundecanoic acid prepared in EtOH for 16 h at 25 °C. The mixture was washed several times with deionized water via centrifugation at 13,000 rpm for 10 min, followed by redispersion in 50 mM MES buffer (pH 5.0). To activate the groups on the surface of carboxylated SiO_2_@Au–Ag NPs, EDC hydrochloride (2 mg) and sulfo-NHS (2 mg) were added to carboxylated SiO_2_@Au–Ag NPs prepared in 50 mM MES buffer, and the mixture was stirred for 30 min at 25 °C. After removing the supernatant via centrifugation, NPs were dispersed in MES buffer. SiO_2_@Au–Ag NPs were added to the anti-PSA Ab mixture (cat. no. 14803; 1 mg/mL, 150 μL), and the mixture was stirred for 2 h at 25 °C. After centrifugation, anti-PSA Ab-introduced SiO_2_@Au–Ag NPs in 50 mM MES were mixed with ethanolamine (3.2 μL), and the mixture was stirred for 30 min at 25 °C. The mixture was then washed several times with 0.5% bovine serum albumin (BSA) prepared in phosphate-buffered saline (PBS) via centrifugation at 13,000 rpm for 10 min and then redispersed in 0.5% BSA in PBS.

### Synthesis of anti-PSA Ab-conjugated colloid AuNPs

AuNPs (15 nm diameter) were prepared as reported previously [38]. Water (99 mL) and 25 mM HAuCl_4_·3H_2_O (1 mL) were mixed in an Erlenmeyer flask and stirred vigorously for 5 min at 100 °C. Subsequently, 3.3% sodium tricitrate in deionized water was added rapidly to the mixture, and the mixture was stirred for an additional 1 h. Thereafter, the mixture was cooled to room temperature, and its volume was adjusted to 100 mL with deionized water. Subsequently, the pH of the mixture was adjusted to 8.5 using polyvinylpyrrolidone (100 mg).

Thereafter, 2 mM AuNPs (1 mL) in deionized water was mixed with anti-PSA Ab (cat. no. 14803; 1 mg/mL, 200 μL), and the mixture was incubated overnight at 4 °C. After completion of the reaction, the mixture was centrifuged at 17,000 rpm for 15 min for sedimentation and dispersed in PBS containing 0.5% BSA.

### Characterization of SiO_2_@Ag@SiO_2_ NPs

Transmission electron microscopy (TEM) was performed using a LIBRA 120 (Carl Zeiss, Oberkochen, Germany). Field emission-scanning electron microscopy (FE-SEM) was performed using an SU-8010 instrument (Hitachi, Tokyo, Japan). UV–Vis extinction spectra were obtained with an OPTIZEN POP instrument (Mecasys, Daejeon, Korea).

### Preparation of test strips

The test strips comprised a backing card, NC membrane, and absorbent pad (Additional file 1: Fig. S1). After assembling the NC membrane and absorbent pad on the backing card, anti-PSA Ab prepared in PBS (cat. no. 14801; 1 mg/mL) was sprayed on the test line, and the control line was sprayed with goat anti-rabbit IgG prepared in PBS (1 mg/mL) using a dispenser. The strip was dried for approximately 24 h. Next, 0.1% BSA was applied and the assembly was dried for at least 1 day. Finally, the absorbent pad was assembled on the backing card. After cutting the strips to a width of 4 mm, the test strips were prepared.

### Collection of clinical samples

Before starting this study, all clinical samples were obtained with written informed consent, and the study design was approved by the Seoul National University Bundang Hospital (IRB No. B 1711/432–302).

### Analysis of colored test lines in the test strips

To measure the color intensity of the test line on the test strips, images of test strips were captured as 8-byte images using an ImageQuant LAS-4000 (GE Healthcare, Chicago, IL, USA).^31^ Captured images were analyzed using ImageJ ver. 1.53a (National Institutes of Health, Bethesda, MD, USA).

Stability test for SiO_2_@Au–Ag NPs as signal reporters in LFIAnti-PSA Ab-conjugated SiO_2_@Au–Ag NPs were stored in 0.5% BSA in PBS to evaluate stability for 14 days. Every 2 days, stored anti-PSA Ab-conjugated SiO_2_@Au–Ag NPs were mixed with clinical samples spiked with 0.54 ng/mL PSA, and LFIA was performed in three batches. After analysis, the test strip was checked visually, and the color intensity of the test line of the test strip was measured using ImageJ.

## Results and Discussion

### Synthesis of SiO_2_@Au–Ag NPs

**Fig. 1:**
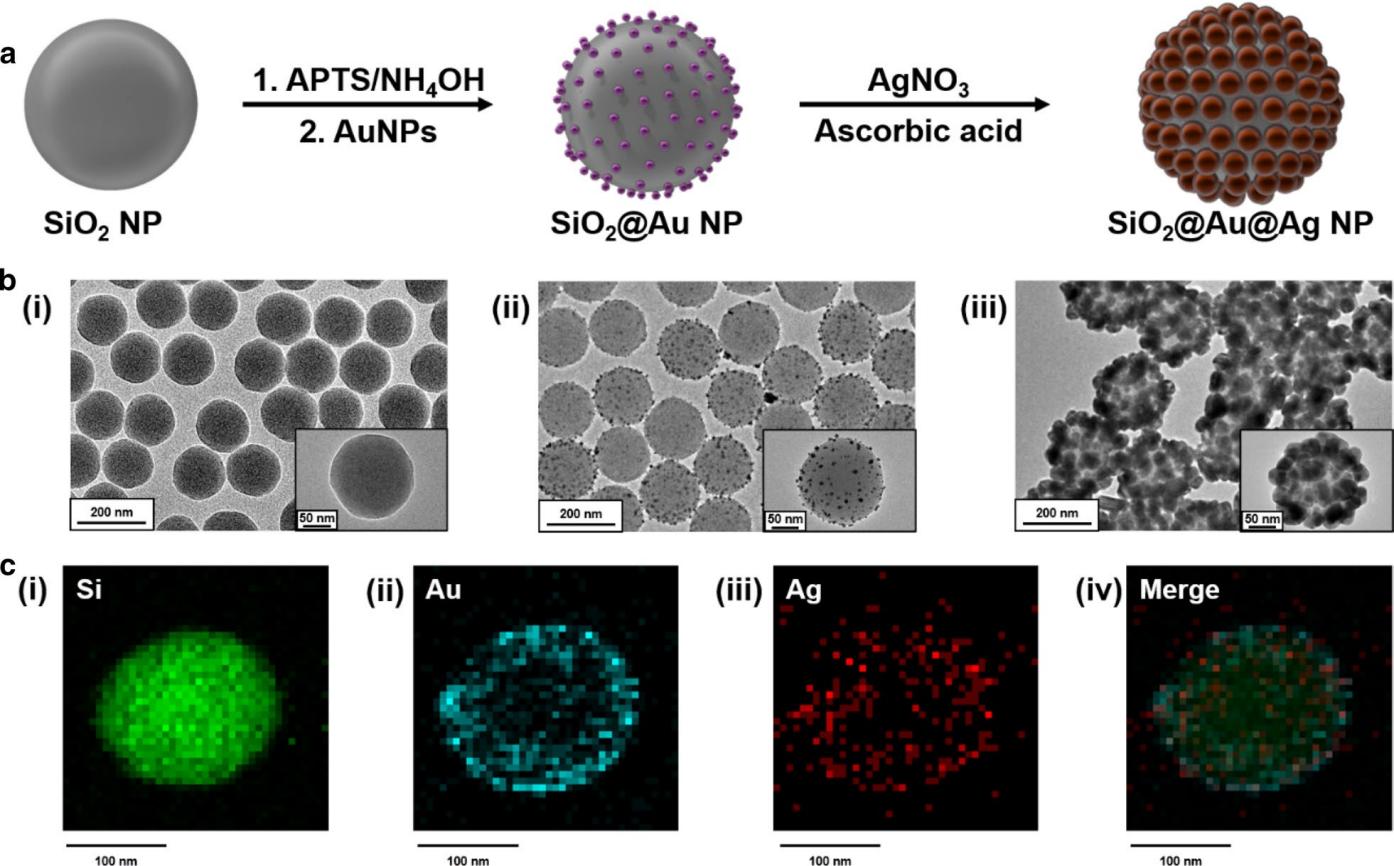
Synthesis of SiO_2_@Au–Ag NPs. a Synthesis procedure for SiO_2_@Au–Ag NPs. **b** Transmission electron microscopy (TEM) images of (i) SiO_2_ NPs, (ii) SiO_2_@Au NPs, and (iii) SiO_2_@Au–Ag NPs. **c** EDX mapping showing each component of SiO_2_@Au–Ag NPs, including (i) Si atoms, (ii) Au atoms, and (iii) Ag atoms. (iv) Overlay image of all elements

**Fig. 2:**
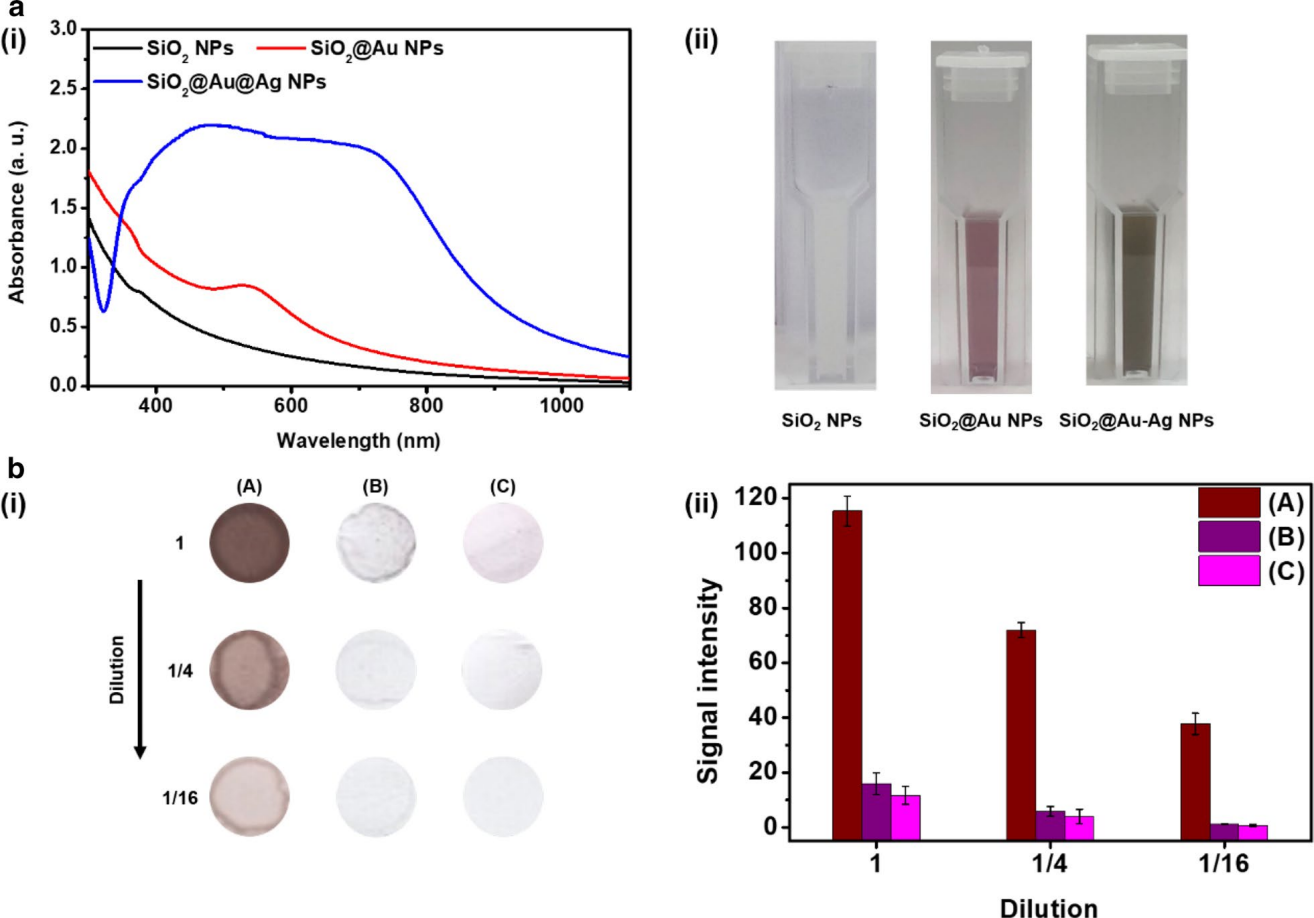
Characterization of SiO_2_@Au–Ag NPs. **a**(i) UV–vis extinction spectra of SiO_2_ NPs, SiO_2_@Au NPs, and SiO_2_@Au–Ag NPs. **a**(ii) Color image of SiO_2_ NPs, SiO_2_@Au NPs, and SiO_2_@Au–Ag NPs in EtOH solution. **b**(i) Scanned color image of the spots formed on the NC membrane (6 mm) with 3 μL suspension containing **A** SiO_2_@Au–Ag NPs, **B** SiO_2_@Au NPs, and **C** colloid AuNPs at different dilutions (1, 1/4, and 1/16). (b(ii)) Signal intensity based on the scattering effect of corresponding-colored spots on the NC membrane. Error bars represent the standard deviations of the means for three batches of analyte measurements

The process used for synthesizing Au–Ag alloy NPs assembled on silica NPs (SiO_2_@Au–Ag NPs) is described in Fig. 1a. SiO_2_ NPs (160 nm) were synthesized using the Stöber method (Fig. 1b(i)) [29]. Subsequently, the NP surfaces were modified to amine groups, and colloid AuNPs (7 nm) were introduced onto the SiO_2_ NPs (SiO_2_@Au NPs, Fig. 1b(ii)). Silver ions on the surface of AuNPs were reduced, yielding SiO_2_@Au–Ag NPs (Fig. 1b(iii)) [32]. The chemical compositions of the SiO_2_@Au–Ag NP were investigated using energy dispersive spectroscopy (EDX) mapping. The locations of the Si, Au, and Ag atoms in the SiO_2_@Au–Ag NPs are shown in Fig. 1c(i–iv). EDX revealed that both AuNPs and AgNPs were located on the surface of silica NPs. These results demonstrated the successful fabrication of SiO_2_@Au–Ag NPs.Next, we investigated the characteristics of the synthesized SiO_2_@Au–Ag NPs. Figure 2a(i) shows the UV–vis absorption spectra for each sample during the synthesis of SiO_2_@Au–Ag NPs. SiO_2_@Au NPs showed unique absorbance peaks at approximately 520 nm (compared with SiO_2_ NPs), and SiO_2_@Au–Ag NPs showed a wide absorbance band (400‒800 nm; compared with SiO_2_@Au NPs) [36]. These results demonstrated that an Ag layer was formed on the surface of AuNPs immobilized on SiO_2_ NPs. Figure 2a(ii) shows color images of SiO_2_ NPs, SiO_2_@Au NPs, and SiO_2_@Au–Ag NPs (1 × 10^10^ particles/mL) dispersed in EtOH. Assembly of NPs is known to induce plasmon vibrations, which cause a color change [39]. Visually, because the band of the finally synthesized SiO_2_@Au–Ag NPs exhibited a wide spectrum of absorbance, it appeared dark brown in EtOH.

We also investigated the scattering effect of SiO_2_@Au–Ag NPs on light in the NC membranes of test strips. Normally, the band on the NC membrane absorbs and scatters light according to the plasmon properties of the signal reporter. As previously reported, a series of two consecutive 1/4 dilutions of SiO_2_@Au–Ag NPs, SiO_2_@Au NPs, and colloid AuNPs (size: 15 nm, λ_abs,max_: 520 nm) were prepared (Additional file 1: Fig. S3). The prepared samples were dropped by spotting on the circular NC membrane (6 mm), and visibility was confirmed based on the distribution of color spots according to NP dilution. First, 3 μL of each sample was dropped onto the NC membrane and dried, and then the NC membrane was scanned. Figure 2b(i) shows an image of colored spots obtained upon applying a dilution (top to bottom). Upon visual analysis, the colored spots faded as the concentration of each sample decreased; when all signal reporters were diluted to 1/16, only the color intensity of SiO_2_@Au–Ag NPs was observed. When comparing the color intensity between all signal reporters, SiO_2_@Au–Ag NPs showed the brightest signal compared with other samples at all dilutions (Fig. 2b(ii)). These results confirmed that SiO_2_@Au–Ag NPs were suitable for use in the LFIA platform owing to their high scattering effect in the NC membrane of the test strips.

### Changes in the color intensity of the test line according to the concentration of PSA in LFIA

**Fig. 3:**
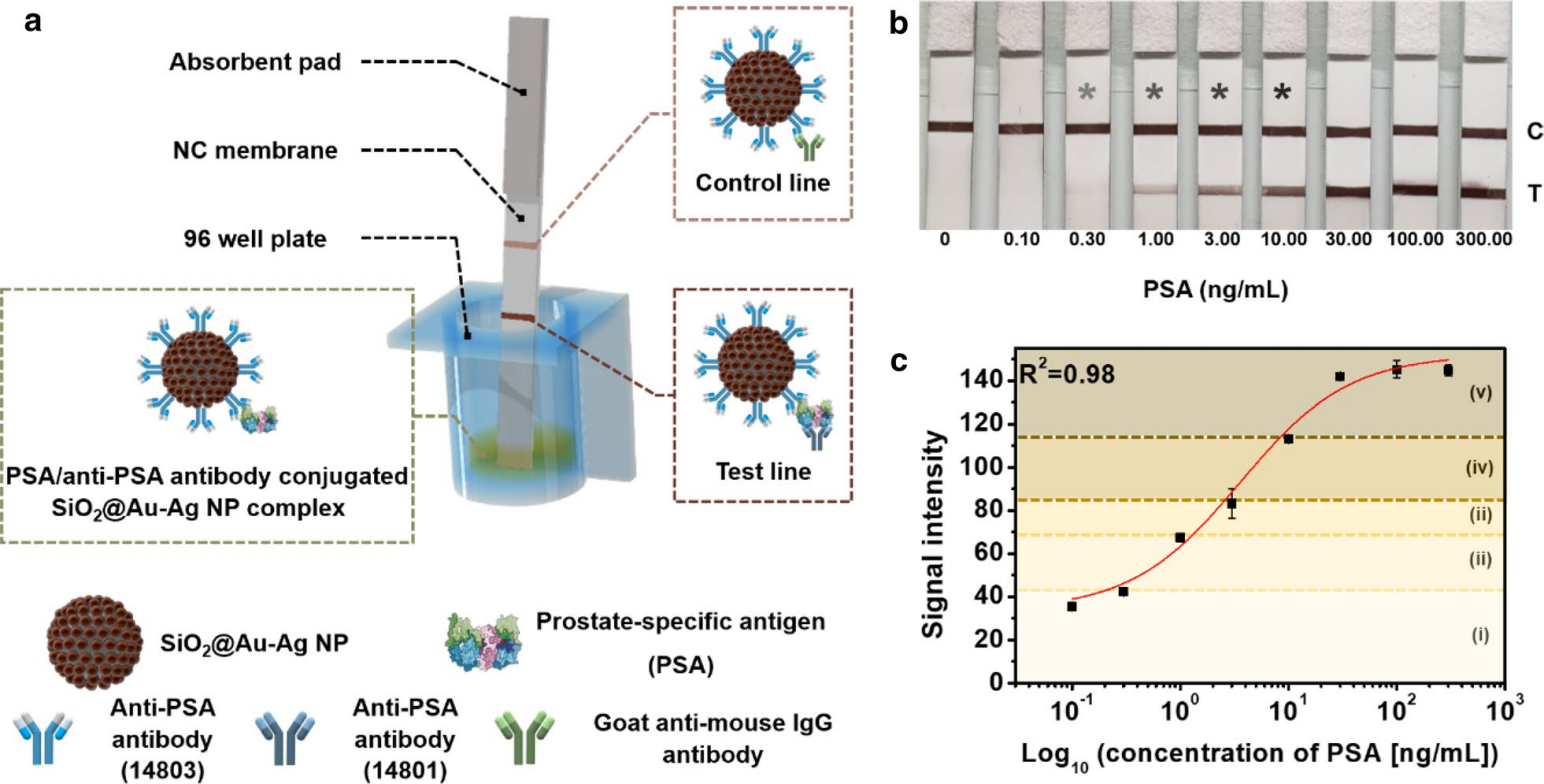
Schematic illustration of PSA detection using the lateral flow immunoassay (LFIA) platform with SiO_2_@Au–Ag NPs as a signal reporter. **b** Color. **c** Schematic of early diagnosis and prognosis detection by measuring the signal intensity [i: no recurrence (PSA < 0.30 ng/mL), ii: recurrence (0.30–1.00 ng/mL PSA), iii: no cancer (1.00–3.00 ng/mL PSA), iv: early-stage disease (3.00–10.00 ng/mL PSA), and v: late-stage disease (PSA > 10.00 ng/mL)]. Error bars represent the standard deviations of the means for three batches of analyte measurements

To utilize SiO_2_@Au-Ag NPs in the LFIA platform, the surface of SiO_2_@Au-Ag NPs was modified at carboxyl groups using 11-mercaptoundecanoic acid. Next, the surface was activated using EDC/sulfo-NHS, and anti-PSA Abs were conjugated onto the surface. The UV-Vis absorption spectrum of anti-PSA Ab-conjugated SiO_2_@Au-Ag NPs was preserved after conjugation of anti-PSA Abs (Additional file 1: Fig. S2). The working principle of the LFIA is shown in Fig. 3a. The test strip comprised an absorbent pad and an NC membrane; after assembling the components, the dipstick method was used for PSA and anti-PSA Ab-conjugated SiO_2_@Au-Ag NPs in 96-well plates. The results showed that PSA and anti-PSA Ab-conjugated SiO_2_@Au-Ag NPs formed complexes in the wells, and test strips were then dipped in the corresponding wells. After 15 min, the test line on the test strip turned dark brown, a phenomenon that could be confirmed visually owing to the interaction between the complex and the capture antibody.

Fig. 3b and c show the results of concentration-specific analysis of PSA using the newly developed LFIA platform. Analysis was performed at PSA concentrations ranging from 0 to 300.00 ng/mL. As the concentration of PSA increased, more immune complexes were formed at the test line, and the brown color of the test line became darker, as expected. At a concentration of 0.30 ng/mL, the test line could be observed with the naked eye (Fig. 3b). Additional file 1: Fig. S4 shows scanning electron microscopy (SEM) images of the test line on test strips (at PSA = 0 ng/mL and PSA = 300.00 ng/mL). SiO_2_@Au-Ag NPs bound to the NC membrane through PSA were clearly different from the test line for 0 ng/mL in SEM. When more PSA antigen-SiO_2_@Au-Ag NPs complexes were captured on the test line, the signal intensity of the test line increased as the concentration of PSA increased (Fig. 3c). Analysis of the calibration curves based on the signal intensity revealed that the limit of detection (LOD) was 0.20 ng/mL. Based on these semiquantitative results and PSA concentrations of 1.00–10.00 and 0.30–1.00 ng/mL, the color intensity of the test line on the test strip was used as a standard for early diagnosis. Specifically, normal status was indicated as a signal in the 1.00–3.00 ng/mL PSA section of the test strip; early-stage disease was indicated as a signal in the 3.00–10.00 ng/mL PSA section of the test strip; and late-stage disease was indicated as a signal at greater than 10.00 ng/mL PSA. In addition, to monitor for recurrence, biochemical recurrence was indicated as a signal in the 0.30–1.00 ng/mL PSA section, whereas no recurrence was indicated as a signal at less than 0.30 ng/mL PSA. As a standard, a band showing different colors dependent on the concentration at the test line was sufficient to distinguish each stage, and the low sensitivity was suitable for use in patient prognosis. For comparison of the detection ability of the SiO_2_@Au-Ag NPs, existing colloid AuNPs conjugated to anti-PSA Ab were evaluated, and the LODs of these two signal reporters were calculated at the same concentration of PSA (Additional file 1: Fig. S5). The band on the test line could be observed at a PSA concentration of 1.00 ng/mL when colloid AuNPs were used as the signal reporter. Moreover, the color intensity difference according to the PSA concentration for each strip using colloid AuNPs was not greater than that of SiO_2_@Au-Ag NPs. The LOD determined from the standard deviation was 0.82 ng/mL for colloid AuNPs. Based on the calculated LODs, SiO_2_@Au-Ag NPs were approximately four times more sensitive than colloid AuNPs and SiO_2_@Au NPs, and these results demonstrated the high performance of the prepared SiO_2_@Au-Ag NPs for PSA detection with LFIA. Additionally, these results demonstrated the applicability of this approach as a standard.

### Clinical validation of LFIA for PSA detection

**Fig. 4:**
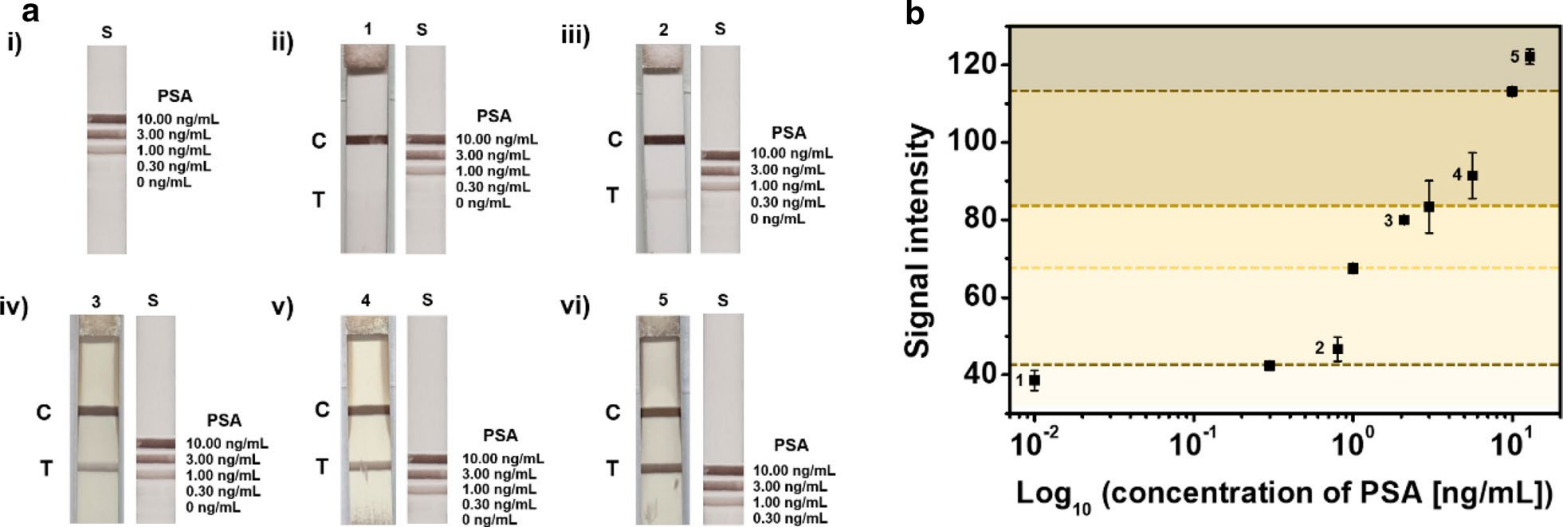
Application of clinical samples with SiO_2_@Au–Ag NPs as a signal reporter by comparison with the standard in LFIA. **a** Color image test line (T) on each test strip (i: PSA standard (S), ii PSA < 0.01 ng/mL (1), iii: 0.80 ng/mL PSA (2), iv: 2.01 ng/mL PSA (3), v: 5.61 ng/mL PSA (4), and vi: 12.84 ng/mL PSA (5). **b** Measurement of signal intensity using clinical samples with the standard in the LFIA platform. Error bars represent standard deviations of the means for three batches of analyte measurements

PSA detection in clinical samples is essential for assessing the performance and practical applicability of the LFIA platform. Therefore, we evaluated the applicability of LFIA with the standard obtained from the previous results using SiO_2_@Au-Ag NPs for PSA detection in clinical samples. Previously, clinical samples (1: < 0.01 ng/mL, 2: 0.80 ng/mL, 3: 2.01 ng/mL, 4: 5.61 ng/mL, and 5: 12.84 ng/mL) were prepared by selecting samples corresponding to different sections of the standard obtained from the above results. When applying actual clinical samples to the LFIA platform, the amount of clinical sample used was 30 μL, which is less than that used for conventional enzyme-linked immunosorbent assays [40]. Clinical samples with PSA concentrations corresponding to each section were used, and PSA samples with PSA concentrations of less than 0.01 ng/mL were used as negative controls. As shown in Fig. 4a, except for the negative control (strip 1), all other PSA-positive samples showed bands on the test line. When measuring the color intensity of the band on the test line for each strip, the color intensities were correlated with the concentration of PSA. Additionally, when visually comparing the results with the color intensity of the standard, we confirmed that the bands produced by the clinical samples were within the appropriate section of the test strip (Fig. 4b). Thus, PSA in clinical samples was visually different from that in negative control samples, even at low concentrations, when using the prepared assay system.

In the LFIA using SiO_2_@Au-Ag NPs, because the visual LOD of the test line was 0.30 ng/mL PSA, a clinical sample with a PSA concentration of 0.32 ng/mL was applied to the LFIA to confirm the visual LOD in clinical samples. Importantly, the intensity at the test line appeared similar to that in the standard but was slightly higher when the results were compared using ImageJ (Additional file 1: Fig. S6). Thus, these results showed that the visual LOD was consistent in clinical samples. In this study, complete quantitative analysis was difficult using the developed LFIA platform. However, the platform was suitable for screening because the stage of progression could be distinguished through analysis of the color intensity during early diagnosis and prognosis [41].

### Reproducibility and stability tests using SiO_2_@Au-Ag NPs in the LFIA platform

**Fig. 5:**
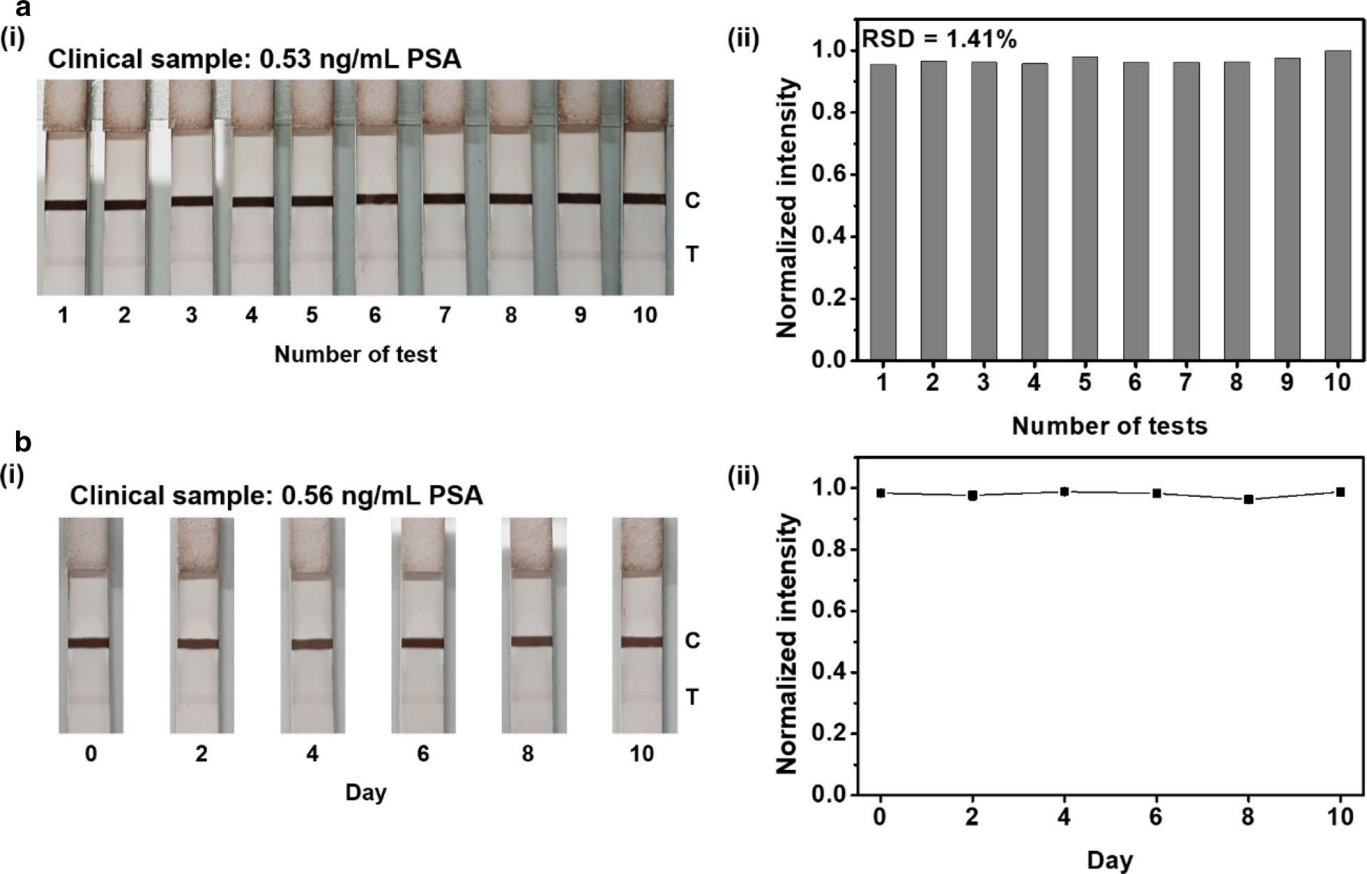
**a** Test of reproducibility using SiO_2_@Au–Ag NPs as a signal reporter with clinical samples (0.53 ng/mL PSA) in LFIA. (i) Color images and (ii) measurement of signal in-tensity. **b** Test of stability using SiO_2_@Au–Ag NPs with clinical sample (0.56 ng/mL PSA) in LFIA. (i) Color image and (ii) measurement of signal intensity. Error bars represent standard deviations of the means for three batches of analyte measurements

To validate our clinical sample analysis, we assessed the reproducibility of LFIA for PSA detection in serum using SiO_2_@Au-Ag NPs and the storage stability of SiO_2_@Au-Ag NPs as the signal reporter for LFIA application. First, the reproducibility test was performed with 10 batches of clinical samples (0.53 ng/mL PSA) simultaneously. As shown in Fig. 5a(i), the color intensity of the test line on the test strip was visually constant for samples with the same PSA concentration. The signal intensity of the test line on each test strip was analyzed using ImageJ (Fig. 5a(ii)), and the relative standard deviation (RSD) of the color intensity of the test line for the 10 batches was 1.41%, indicating high reliability [42]. Therefore, these findings confirmed the reproducible performance of the developed platform for the detection of PSA. Second, storage stability tests were conducted using SiO_2_@Au-Ag NPs stored in 0.5% BSA for the evaluation of clinical samples (0.56 ng/mL PSA) with the developed LFIA platform. Visually, the color of the test line in the test strip was almost constant until approximately 10 days (Fig. 5b(i)). Similar results were observed upon analysis of the signal intensity of the test line using ImageJ (Fig. 5b(ii)). The RSD of the signal intensity was 0.96% for day 10, indicating high reliability. Based on these results, we concluded that the developed platform had high reproducibility in terms of performance, even after storage for 10 days.

## Conclusion

Herein, we described a visual LFIA with SiO_2_@Au-Ag NPs as detection signal reporters for semiquantitative detection of PSA in clinical samples. SiO_2_@Au-Ag NPs exhibited a broad absorption spectrum of 400–800 nm ​​as Au-Ag NPs were successfully assembled onto SiO_2_ NPs, and the scattering effect was significantly superior to that of SiO_2_@Au NPs and colloid AuNPs. In addition, in the LFIA platform for PSA detection, we observed a color change that depended on the PSA concentration, and these results were used as a standard for semiquantitative analysis. Furthermore, PSA could be visually detected in clinical samples, and the color intensity corresponded to the PSA concentration, providing a platform for semiquantitative analysis and enabling diagnosis and prognosis. In addition, in reproducibility tests using clinical samples, the RSD for 10 batches was 1.41%, indicating high reproducibility, and the results of stability tests using SiO_2_@Au-Ag NPs in 0.50% BSA solution showed that the signal was almost constant after storage for 10 days. Overall, these results showed the possibility of application of this approach for early diagnosis and prognosis by comparison with the standard. The developed LFIA platform showed limited ability for quantitative analysis; however, the system could be used for quantitative analysis of more advanced particles. Furthermore, our findings demonstrated the applicability of the method for analyzing other clinical samples that require semiquantitative analysis.

## Supplementary Information

**Additional file 1**: **Figure S1**. Schematic illustration of the preparation of test strip. **Figure S2**. UV-vis absorption spectra of SiO2@Au-Ag NPs and anti-PSA conjugated SiO2@Au-Ag NPs. **Figure S3**. Characterization of colloid AuNPs. (a) Transmission electron microscopy (TEM) image. (b) UV-vis extinction spectra. **Figure S4**. Scanning electron microscope (SEM) images of the test line on the test strip. (a) the test line on the test strip with PSA 0 ng/mL, (b) the test line on the test strip with PSA 300.00 ng/mL. **Figure S5**. Detection of various concentrations of PSA using colloid AuNPs as a signal reporter in LFIA. (a) Color images and (b) measurement of signal intensity. Error bars represent the standard deviations of the means for three batches of analyte measurements. **Figure S6**. Application of clinical samples (0.32 ng/mL PSA) with SiO2@Au-Ag NPs as a signal reporter by comparison of test strips detecting 0.3 ng/mL PSA in LFIA. (a) Color images and (b) measurement of signal intensity. Error bars represent the standard deviations of the means for three batches of analyte measurements.

## Data Availability

All data generated or analyzed during this study are included in this manuscript and its supplementary material.

## Abbreviations

PCa: Prostate cancer
RT: Radiation treatment
PSA: Prostate-specific antigen
BCR: Biochemical recurrence
LFIA: Nanoparticle
NC: Nitrocellulose
BSA: Bovine serum albumin
PBS: Phosphate-buffered saline
TEM: Transmission electron microscopy
EDX: Energy dispersive spectroscopy
LOD: Limit of detection
RSD: Relative standard deviation
