# Supplementary material for: Au–Ag assembled on silica nanoprobes for visual semiquantitative detection of prostate-specific antigen
Source: J Nanobiotechnology. 2021 Mar 12;19:73. doi: 10.1186/s12951-021-00817-4 (PMC7953718; doi:10.1186/s12951-021-00817-4)
Supplement: Supplementary file 1 — Additional file 1: Figure S1. Schematic illustration of the preparation of test strip. Figure S2. UV-vis absorption spectra of SiO2@Au-Ag NPs and anti-PSA conjugated SiO2@Au-Ag NPs. Figure S3. Characterization of colloid AuNPs. (a) Transmission electron microscopy (TEM) image. (b) UV-vis extinction spectra. Figure S4. Scanning electron microscope (SEM) images of the test line on the test strip. (a) the test line on the test strip with PSA 0 ng/mL, (b) the test line on the test strip with PSA 300.00 ng/mL. Figure S5. Detection of various concentrations of PSA using colloid AuNPs as a signal reporter in LFIA. (a) Color images and (b) measurement of signal intensity. Error bars represent the standard deviations of the means for three batches of analyte measurements. Figure S6. Application of clinical samples (0.32 ng/mL PSA) with SiO2@Au-Ag NPs as a signal reporter by comparison of test strips detecting 0.3 ng/mL PSA in LFIA. (a) Color images and (b) measurement of signal intensity. Error bars represent the standard deviations of the means for three batches of analyte measurements. [file 12951_2021_817_MOESM1_ESM.docx]

**[Supplementary Material]**

**Au-Ag Assembled on Silica Nanoprobes for Visual Semi-Quantitative Detection of Prostate-Specific Antigen**

Hyung-Mo Kim,^1^ Jaehi Kim,^1^ Jaehyun An,^1^ Sungje Bock,^1^ Xuan-Hung Pham,^1^ Kim-Hung Huynh,^1^ Yoonsik Choi,^2^ Eunil Hahm,^1^ Hobeom Song,^3^ Jung-Won Kim,^3^ Won-Yeop Rho,^4^ Dae Hong Jeong,^2*^ Ho-Young Lee,^5^* Sangchul Lee^6^* and Bong-Hyun Jun^1^*

**
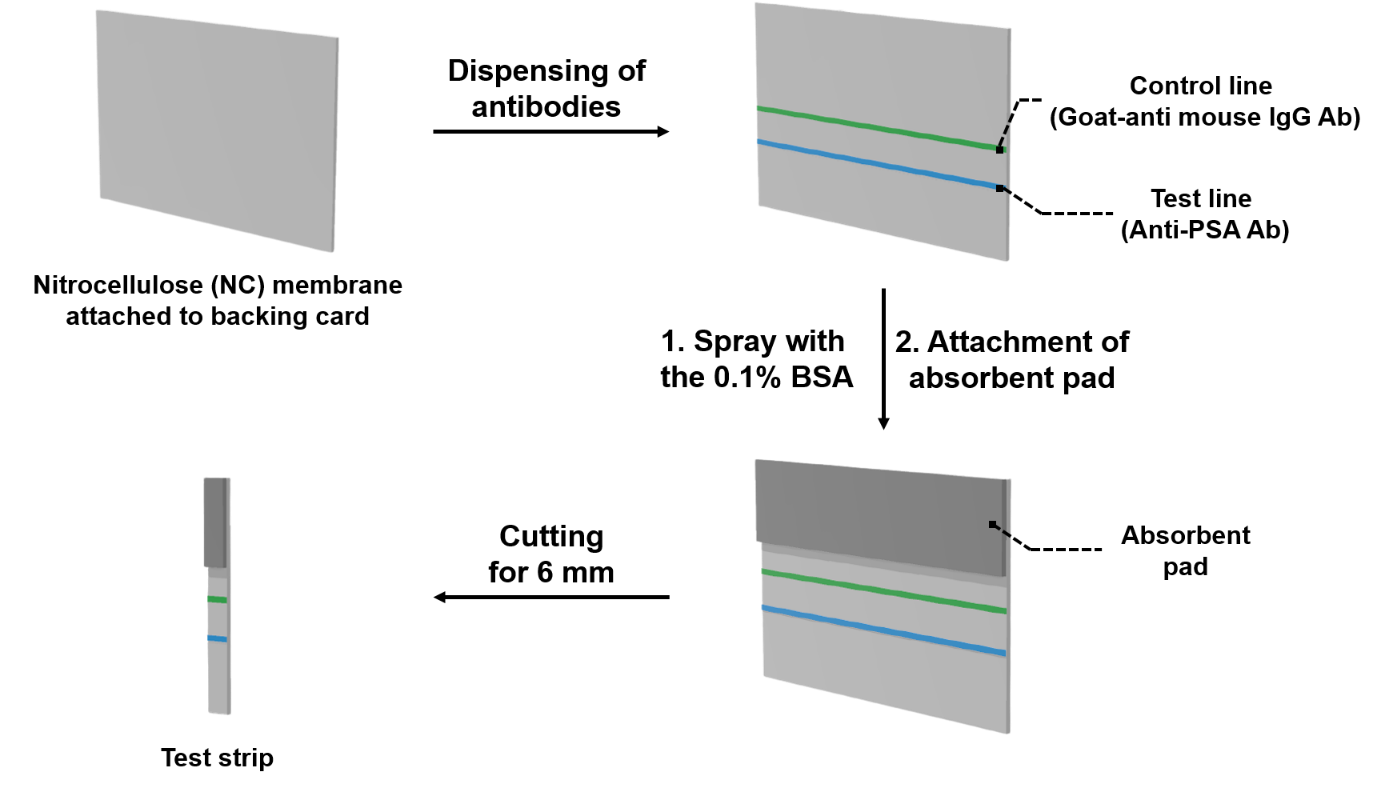
**

**Fig. S1.** Schematic illustration of the preparation of test strip.

**
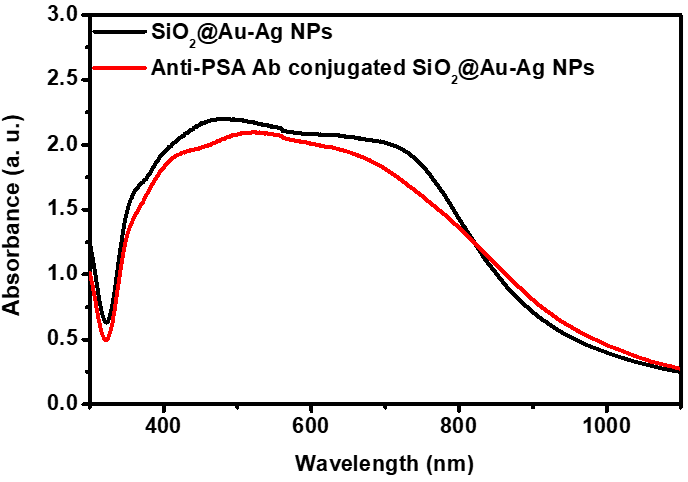
**

**Fig S2.** UV-vis absorption spectra of SiO_2_@Au-Ag NPs and anti-PSA conjugated SiO_2_@Au-Ag NPs.


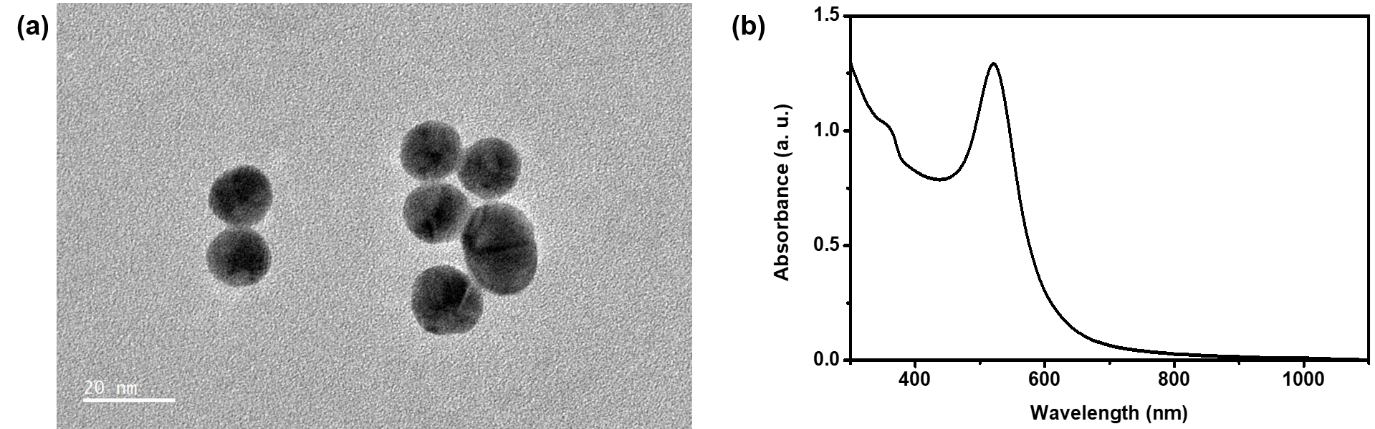


**Fig. S3.** Characterization of colloid AuNPs. (a) Transmission electron microscopy (TEM) image. (b) UV-vis extinction spectra.

**
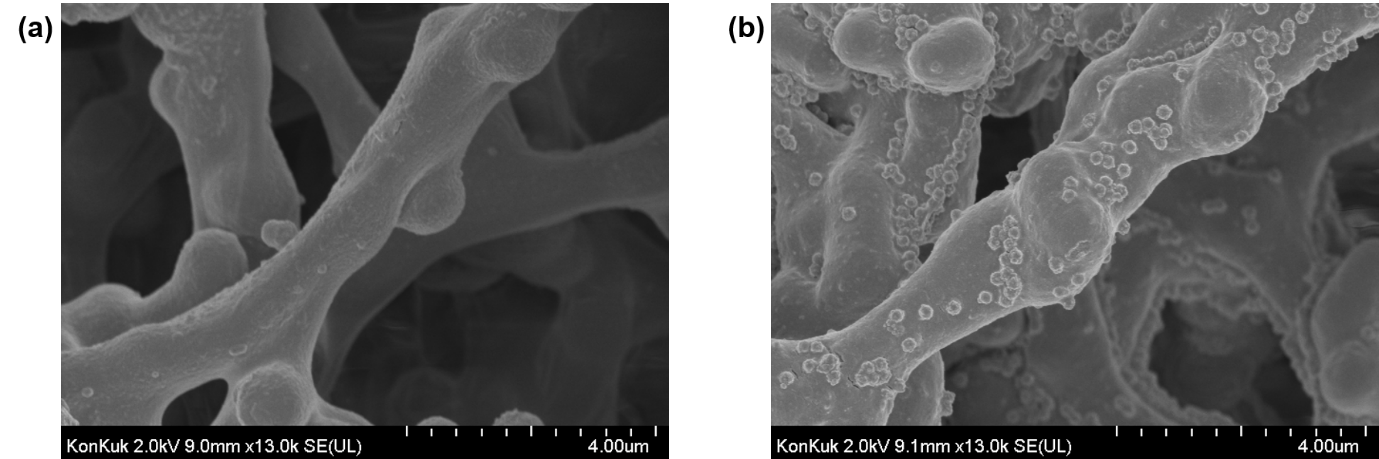
**

**Fig. S4.** Scanning electron microscope (SEM) images of the test line on the test strip. (a) the test line on the test strip with PSA 0 ng/mL, (b) the test line on the test strip with PSA 300.00 ng/mL.


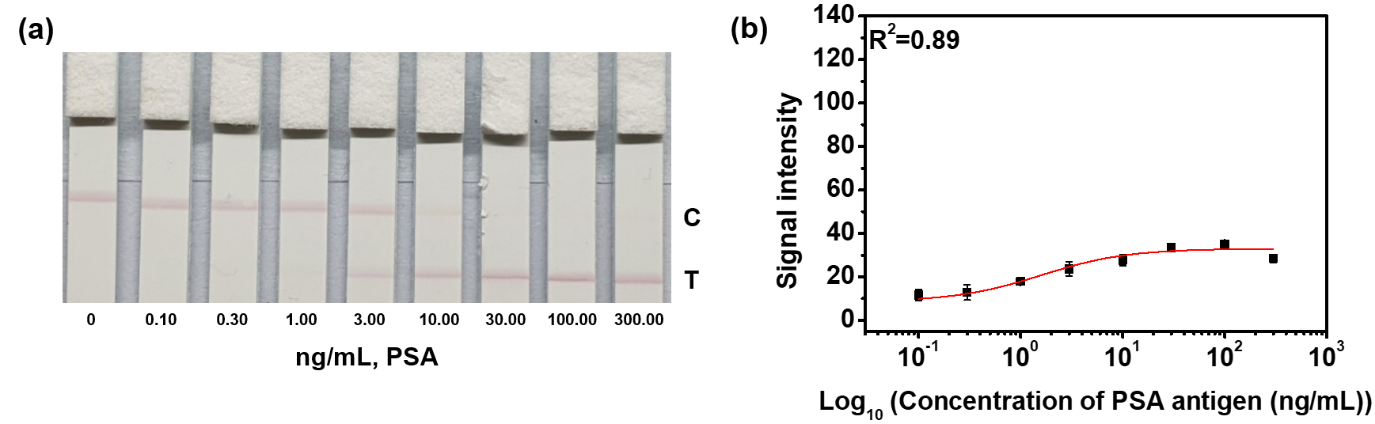


**Fig. S5.** Detection of various concentrations of PSA using colloid AuNPs as a signal reporter in LFIA. (a) Color images and (b) measurement of signal intensity. Error bars represent the standard deviations of the means for three batches of analyte measurements.


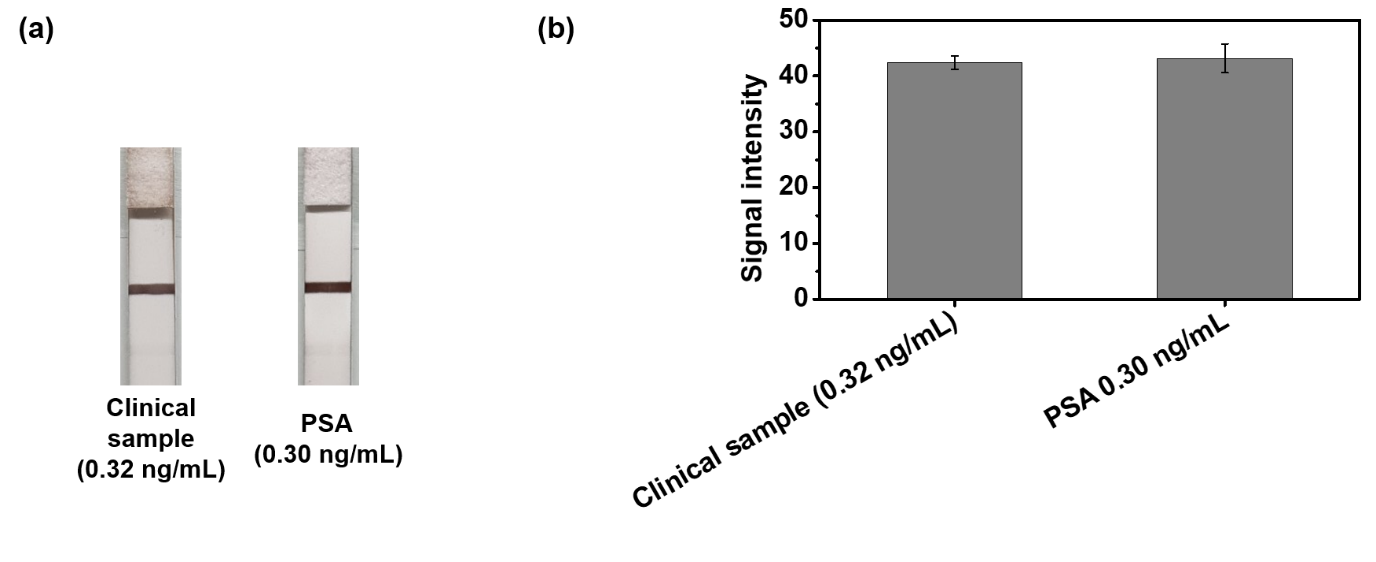


**Fig. S6.** Application of clinical samples (0.32 ng/mL PSA) with SiO_2_@Au-Ag NPs as a signal reporter by comparison of test strips detecting 0.3 ng/mL PSA in LFIA. (a) Color images and (b) measurement of signal intensity. Error bars represent the standard deviations of the means for three batches of analyte measurements.
